# Simulating online and offline tasks using hybrid cheetah optimization algorithm for patients affected by neurodegenerative diseases

**DOI:** 10.1038/s41598-025-93047-9

**Published:** 2025-03-15

**Authors:** Ramkumar Sivasakthivel, Manikandan Rajagopal, G. Anitha, K. Loganathan, Mohamed Abbas, Amel Ksibi, Koppula Srinivas Rao

**Affiliations:** 1 Department of Computer Science, School of Sciences, Christ University, Bengaluru, Karnataka India; 2 Department of Lean Operations and Systems, School of Business and Management, Christ University, Bengaluru, Karnataka India; 3 Department of Computer Science and Engineering, Amrita School of Computing, Amrita Vishwa Vidyapeetham, Chennai, Tamil Nadu India; 4 Department of Mathematics and Statistics, Manipal University Jaipur, Jaipur, Rajasthan 303007 India; 5 Central Labs, King Khalid University, P.O. Box 960, AlQura’a, Abha Saudi Arabia; 6 Electrical Engineering Department, College of Engineering, King Khalid University, Abha, 61421 Saudi Arabia; 7 Department of Information Systems, College of Computer and Information Sciences, Princess Nourah bint Abdulrahman University, Riyadh, Saudi Arabia; 8 Department of Computer Science and Engineering, MLR Institute of Technology, Hyderabad, Telangana India

**Keywords:** Brain-Computer interface, Welch power spectral density, Upper motor neuron, Cheetah optimization algorithm, Lower motor neuron, Radial basis function, Common Spatial pattern, Neuroscience, Engineering

## Abstract

Brain-Computer Interface (BCI) is a versatile technique to offer better communication system for people affected by the locked-in syndrome (LIS).In the current decade, there has been a growing demand for improved care and services for individuals with neurodegenerative diseases. To address this barrier, the current work is designed with four states of BCI for paralyzed persons using Welch Power Spectral Density (W-PSD). The features extracted from the signals were trained with a hybrid Feed Forward Neural Network Cheetah Optimization Algorithm (FFNNCOA) in both offline and online modes. Totally, eighteen subjects were involved in this study. The study proved that the offline analysis phase outperformed than the online phase in the real-time. The experiment was achieved the accuracies of 95.56% and 93.88% for men and female respectively. Furthermore, the study confirms that the subject’s performance in the offline can manage the task more easily than in online mode.

## Introduction

**Fig. 1:**
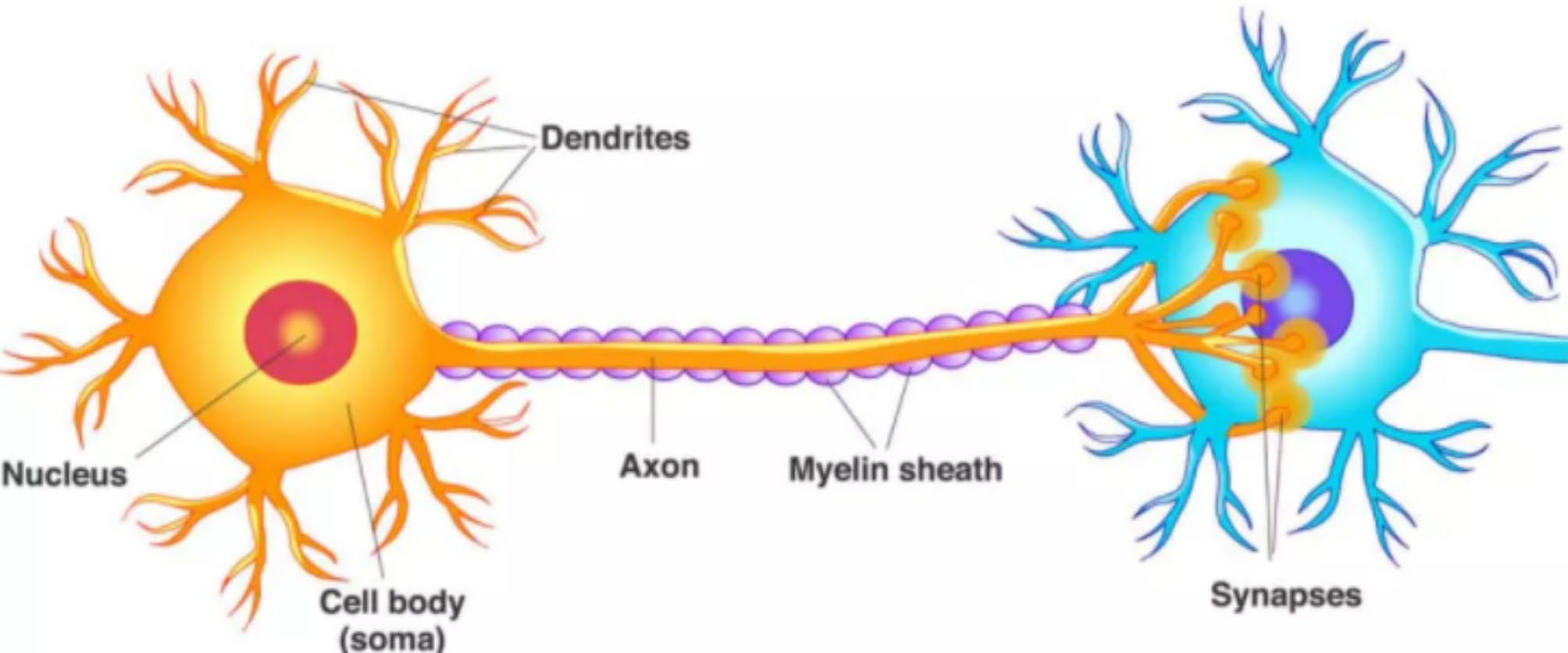
Structure of Neuron.

A neuron is an electrochemical processing unit that processes the signal whenever sufficient input is reached^1^. A neuron consists of three major components they are dendrites, soma, and axon. Neurons receive all the input signals from dendrites. Soma receives all the input signals from the dendrites and sums all the input signals. When a sufficient threshold was attained, the neuron started to fire and connected to the next component called an axon. Axon receive the signals from the soma and connect to the next neurons in the connection path is called Neuronal communication^2–4^ which was shown in Fig. 1.

**Fig. 2:**
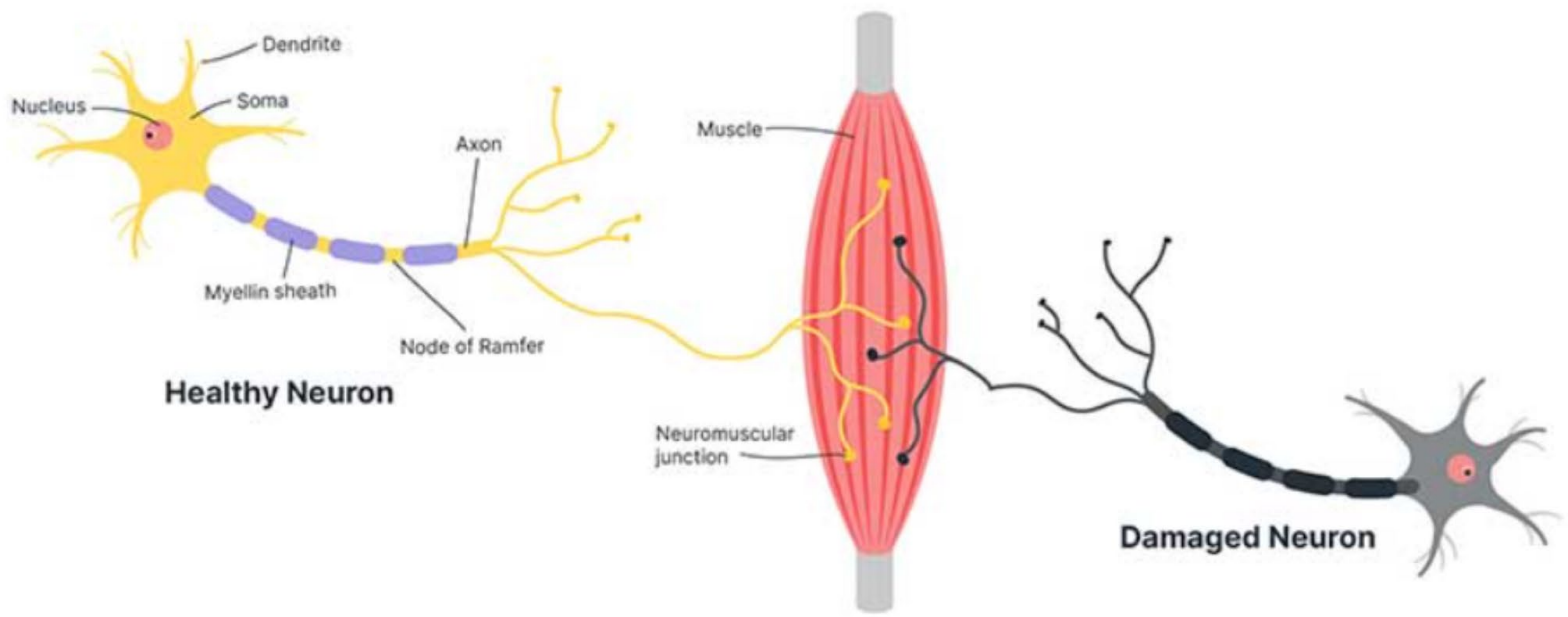
Healthy vs. Damaged Neuron.

Neurodegenerative diseases like Progressive supranuclear palsy, Ataxia, Multiple sclerosis, Multiple System Atrophy, Parkinson’s disease, Motor Neuron Disease (MND), and Alzheimer’s disease will affect the Upper (UMN) and Lower Motor Neuron (LMN) and cause weakness in the muscles and degenerates the neurons present in the communication paths. This neural degenerative disease damages Neuronal communication and slow down our body activities like movement, balance, heart function, breathing and even talking. The difference between the healthy and affected neurons is depicted in Fig. 2. The normal communication between the muscles was disrupted, and eventually, the brain lost all voluntary control due to damage in the neural pathways^5,6^.

In this condition, BCI plays a vital role in the communication to translate the signals into patterns. So there was a great demand and need for BCI to assist the person with motor neuron diseases. It was an intermediate system used to translate the control signals into commands using the trained patterns to operate connected external devices. Currently, most of the researchers are focusing on Electroencephalography techniques to design the BCI, because EEG is one of the non-invasive methods to identify brain patterns uniquely^7–9^. EEG was a method to measure the electrical activities of the brain by placing the electrodes on the scalp non-invasively. Every activity that happens in the brain produces different patterns of signals^10^. By combining these two different technologies, developing the assistive devices for the person with UMN and LMN was very easy. By implementing these techniques, the MND-affected persons can able to fulfill their own needs without other help. It makes the disabled person complete their basic needs without assistance using Speech Synthesizer^11–14^, Keyboard and Mouse Controller^15^, Game Controller^16^, Mouse Controller^17–19^, Hand Controller Devices^20^, Mobile Phone Operators^21^, Facial Expression Detector^22^ Facial Expression Detector^23^ audio speller^24^, Manipulator Robot^25^ and Auditory Brain-Computer Interface^26^. This Study discussed the possibilities of designing a simplified three electrode system to enhance classification accuracy by implementing a hybrid optimization approach and facilitate real world applications in assistive technology to address limited usability in existing BCIs with high complexity. Through this study, we investigate to design and development four states of BCI in offline and online modes through Welch PSD features with a Hybrid Feed Forward Neural Network Cheetah Optimization Algorithm (FFNNCOA).

This remaining part of the manuscript is structured as follows. Section two reviews the related works. The experiment setup and data collection are discussed under section three. The methodology of the proposed work is expressed in the fourth section. Results and evaluation are discussed in the fifth section. Finally, last section concludes the work with future study.

## Related works

Recently several studies using BCIs have been conducted to determine how the brain signals convert to control signals and powered the external devices. Some of the most important studies using advanced feature extraction techniques with hybrid optimization algorithms and standalone classifier models are given below. Ban, et al., have developed a car controller by translating the brain signals to control the smart car by extracting the signals from 12 subjects and achieved an accuracy of 97.65% in online mode^27^. Pawar and Dhage have designed aEEG based speech decoding system for five tasks from 8 subjects and obtained an accuracy of 63.67% with pruning and 57.13% without pruning^28^. Zhu, et al. have proposed the motor imagery classifier to determine the signals produced by the subjects using low-rank representation and B2DDLPP features and obtained an accuracy of 82.27% and 72.26%^29^. Li et al., have designed EEG-based seizure detection systems using DNN and gathered an accuracy of 93.1%^30^. Das and Pachori have developed BCI to recognize the MI tasks performed by the subjects using Multivariate Iterative Filtering and CSP features trained with an LDA classifier and obtained an accuracy of 83.18% and 84.44%^31^. Shi et al., have proposed a advanced BCI to determine the MI tasks generated by the subjects using CSP features trained with three different classifiers and obtained an accuracy of 90–97%^32^.

Smith et al. have developed a intelligent system to detect motor imagery tasks performed by the subjects. Obtained signals were decomposed by using Turnable Q Wavelet Transform and feature extracted with statistical methods and classified with LS-SVM and Radial Basis Function (RBF). From the study, they obtained an accuracy of 99.78% for the RBF classifier^33^. Pawan and Dhiman have designed a EEG-based BCI to establish the MI signals by using modified Binary Grey Wolf Optimization features classified with KNN classifier and obtained accuracy of 92.86% and 91.53%^34^. Jakub Kurczak et al. have developed an EEG-based emotion detection system to determine three different emotions namely neutral, positive, and negative tasks and obtained a classification accuracy of 89%^35^. Tong et al. have designed an EEG-based BCI to determine the pronunciation and MI tasks executed by the participants using CSP features with an adaptive Riemannian Distance classifier and acquired the highest classification accuracy of 90%^36^. Meng et al. have designed EEG-based BCI to classify the signal using CSP features with an SVM classifier and acquired an average classification accuracy between 84.08% and 89.33%^37^.

Pan and Zheng have designed an emotion detection system using power spectral density features with CNN models and achieved an accuracy of 70.34% and 66.50% for FBCCNN and FBSCNN^38^. Bao et al., have developed an EEG-based emotion recognition system using differentiated entropy features with generative adversarial networks and obtained an accuracy of 92.50% and 82.30% for real EEG signals and artificial samples^39^. Kwonet al., have deliberated an EEG-based BCI to identify the subject performances by applying a Deep Convolution Neural Network from 54 subjects for two tasks to identify classification accuracy in terms of subject-dependent and subject-independent^40^. Shi et al., have designed an EEG-based recognition system for rehabilitated individuals by applying the adaptive auto-regressive and CSP features trained with LDA, Bayesian, and common space classifier and achieved an accuracy level above 90.00%^41^. Mishchenko, et al., have designed non-invasive robotic prosthetic systems from 12 subjects using PSD, Energy, and FFT features trained with LDA and SVM classifiers and achieved an accuracy of 80–90% for six different states^42^. AlQattan et al., have designed a sign recognition system using EEG signals for ALS patients by implementing entropy features with SVM and LDA classifier and obtained an accuracy of 76.00% and 75.00%^43^ I. Abidi et al. have developed a taste identification system using the EEG signals by applying the energy, and wavelet entropy features with the LDA classifier and achieved an accuracy of 98.00%^44^. Murugappan et al. have modeled an emotion detection system using statistical features with KNN and LDA classifiers and achieved the highest accuracy of 79.17% from twenty subjects belonging to the age group between 21 and 39^45^.

### Motivation and research gap

From the literature survey, we determined that most of the studies used conventional feature extraction methods with conventional classifiers and obtained average classification accuracy. So in this study, we planned to change the conventional classifier and implement the hybrid optimized bio-inspired algorithms to classify the tasks performed by the subjects in offline and real-time mode in the closed indoor environment.

### Highlights of the work

Some of the important contributions made in the study are given below.

1. Eighteen subjects were involved in the study and also data collected from the subjects.
2. Nine subjects from each male and female were involved in the research.
3. Result analyses were done for online and offline mode.
4. Neural Network model combined with a bio-inspired algorithm to find the hybrid and optimized solution.
5. A real-time study was conducted in an indoor environment.

## Experimental setup and data collection

### Subjects with experimental environment

The experiment involved eighteen subjects, evenly divided between nine males and nine females. All the individuals who took part in the experiment were belonged to the age groups of 20 to 27. Before collecting the data, all subjects involved in the experiment were informed to read and sign the consent form. All participants took part as volunteers, so no payment was provided. All the subjects were trained well before extracting the signals. At the time of collecting the signal, the room was cleaned neatly, the subjects were free from illness, and actively took part in the experiment. All participants were right-handed habited individuals with clean eyesight. Subjects were actively participated in the experiment in both offline and online mode.

### Timeline, stimulus and task description

Our study follows three phases in timeline structure they are: Training Phase before signal collection (45 min to explaining the procedure and conditions in task wise and trials), Offline training phase ( 5 s of data collection by imagery task displayed on a monitor in terms of words for the directions like left, right, forward and stop. Subjects who were involved in the study were requested to perform corresponding tasks during data collection for individual trials) and the Online training phase ( 5 s of data collection by imagery tasks randomly on a monitor in terms of words for the directions like left, right, forward and stop).

### Data acquisition

**Fig. 3:**
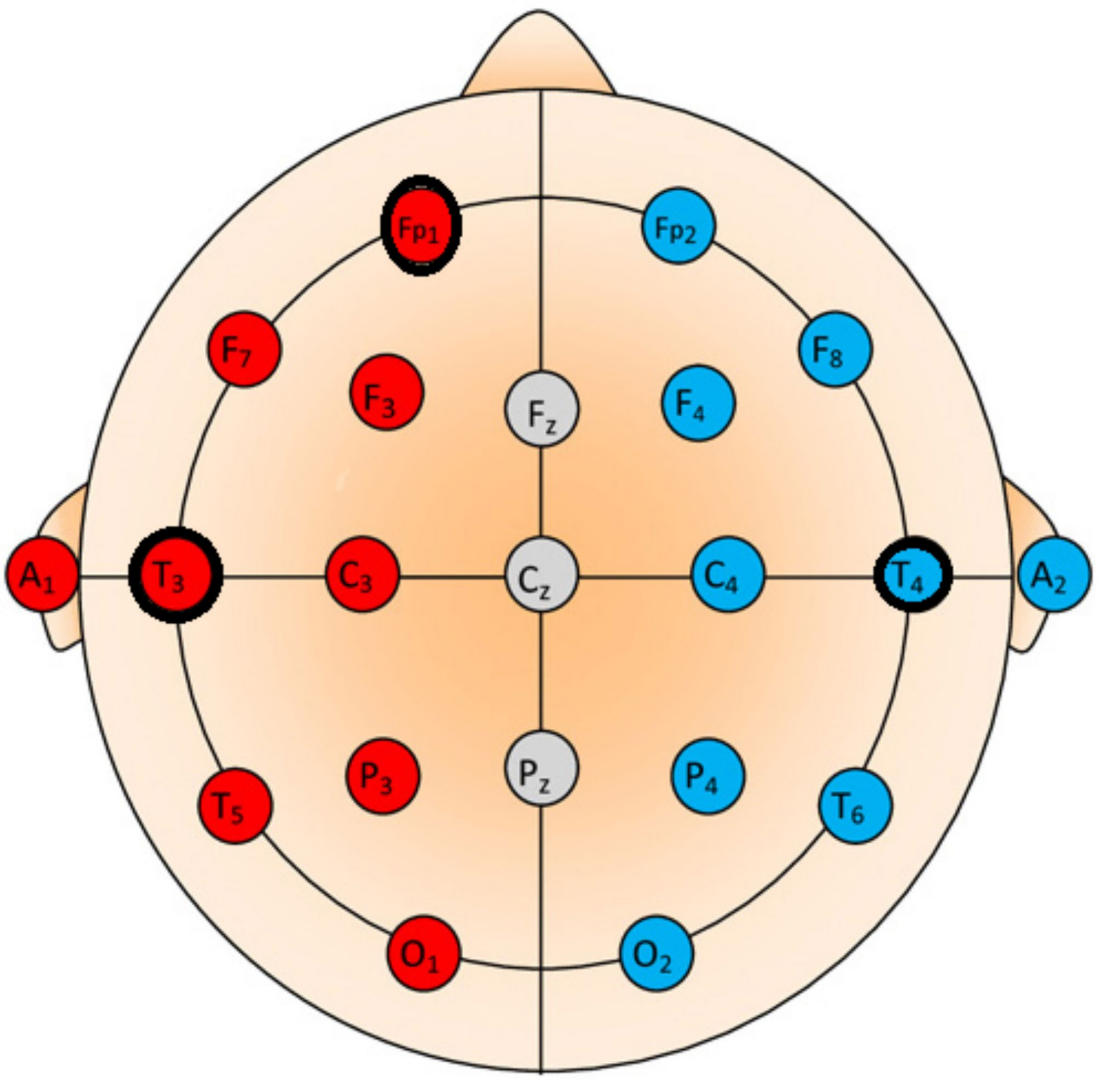
Electrode Placement Implemented in this study.

**Fig. 4:**
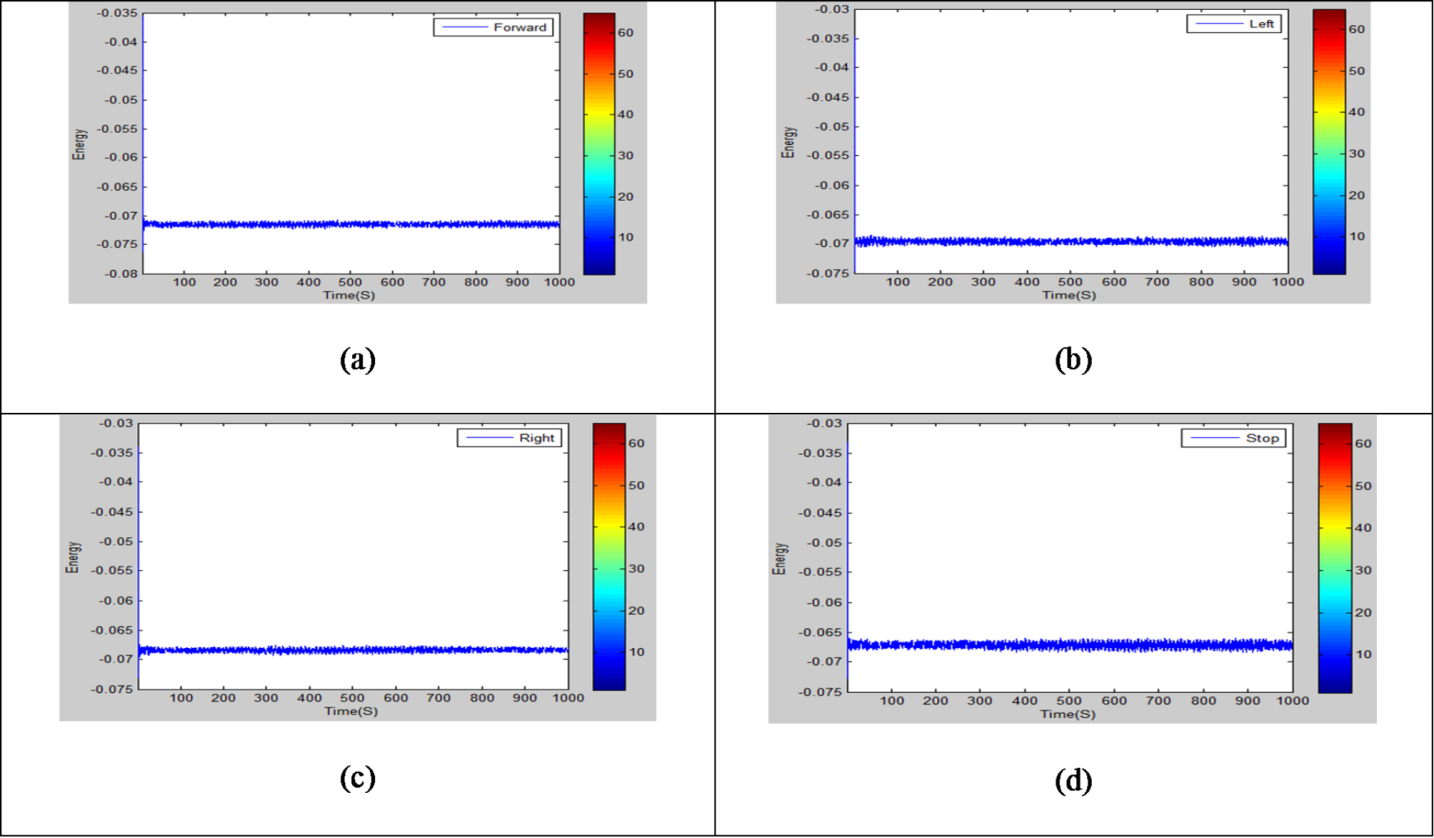
Raw EEG signals were gathered from the subject for tasks like (a) Forward, (b) Left, (c) Right, and (d) Stop in offline mode.

Three electrodes were used to collect the signals from volunteers using Analog Digital Instrument which is shown in Fig. 3. The electrodes present in positions T3 and T4 were used to collect the signals generated by the subjects to capture signals associated with Motor Imagery Tasks. The third electrode FP1 was placed above the left eyebrow to improve frontal lobe activity signal for motor functions and cognitive during signal acquisition which was illustrated in Fig. 3. Participants were performing four imagery tasks without vocalizing the words constantly for four tasks in offline mode and randomly in online mode for five seconds per trial. The recorded data was pre-processed using a notch filter with 50HZ to remove the noise. Signals acquired for each task per sample trial are shown in the Fig. 4. It depicts that the moving forward, turn left, turn right and stop commands in offline setup.

### Feature extraction

During this study, the Welch Power Spectral Density (W-PSD) method was applied to determine the features. It is one of the PSD methods to evaluate the spectrum from the signal. W-PSD determines the power spectra by categorizing the time signal into the continuous block and structuring the periodogram for each continuous block and averaging each successive block^46,47^. It was introduced by the scientist Peter D. Welch. It was one of the improved versions of a standard periodogram and Barlett’s method. This method determines the power spectrum from the obtained signals and reduces the noise at the time of modifying the signals into frequency resolution^48,49^. To convert the extracted signals into features using W-PSD, the following steps were followed.

Step 1: Divide the data sequences into several segments K.

1 $$\:x\left[0\right],x\left[1\right],\dots\:.\:x\left[N-1\right]$$

$$\begin{aligned} & \:\text{S}\text{E}\text{G}1:\:x\left[0\right],x\left[1\right],\dots\:.\:x\left[M-1\right]\\ &\:\text{S}\text{E}\text{G}2:\:x\left[S\right],x\left[S+1\right],\dots\:.\:x\left[M+S-1\right]\\ &\quad\quad \quad \quad \cdot\\&\quad \quad\quad \quad \cdot\\ &SEG K:x\left[ {N - M} \right], \, x\left[ {N - M + 1} \right], \ldots \ldots \ldots x\left[ {N - 1} \right]\\ \end{aligned}$$

Where $\:x\left[0\right],x\left[1\right],\dots\:.\:x\left[N-1\right]$ represent the data sequences, M indicates the block, SEG represents the individual segments, S characterizes the shift point between the blocks and K represents a number of blocks^50^.

Step 2: DFT is applied to each block to windowing the gathered signals at some frequency(v).

2 $$\:v=\frac{i}{M}with\:\left(\frac{M}{2}-1\right)\le\:i\le\:\frac{M}{2}$$

3 $$\:{X}_{K}\left(v\right)={\sum\:}_{m}x\left[m\right]w\left[m\right]exp\left(-j2\pi\:vm\right)$$

From the above equation, $\:m$ can be calculated by using the equation.

$$\:m=\left(k-1\right)\:S,\dots\:\dots\:.M+\left(k-1\right)\:S-1$$

Where $\:w\left[m\right]$ indicates the window function.

Step 3: From Step 2 by applying DFT, modified periodogram raw value $\:{P}_{K}\left(f\right)$obtained for every step segment. The mathematical model to represent this equation is mentioned below.

4 $$\:{P}_{K}\left(v\right)=\frac{1}{W}{\left|{X}_{K}\left(v\right)\right|}^{2}$$

From the Eq. (4),$\:{X}_{K}\left(v\right)$ represented the Fourier Transform values and $\:{P}_{K}\left(v\right)$ indicates the periodogram value^46^.

Step 4: From step 3, calculate the periodogram average value obtained from each block to determine the W-SPD. The mathematical model to estimate the W-PSD is expressed below.

5 $$\:{S}_{x}\left(v\right)=\frac{1}{K}\sum\:_{k=1}^{K}{P}_{K}\left(v\right)$$

Where,$\:M$ indicates the length of blocks, $\:S$ represents the number of shifts between the blocks;$\:K$ represents the number of blocks and $\:v$ indicates the PSD variables^49,50^.Data collected from the different subjects were applied to select the best features to evaluate the tasks. From each trial 22 features were selected and repeated for ten trials for four tasks. Forty features were collected from each subject and overall 720 trails features were obtained and trained with the FFNNCOA algorithm to estimate the optimal classification accuracy.

## Proposed methodology of FFNNCOA

A novel hybrid classification model was proposed in this study. It is a hybrid model with the combination of Feed Forward Neural Networks (FFNN) to handle essential EEG data from non-linear data structures with Cheetah Optimization Algorithm (CAO) to overcome the conventional boundaries of back propagation methods. This methodology reduces the training time and improved classification accuracy. These combinations make sure better adaptability, higher classification accuracy and faster convergence for designing BCI in real time environment. This section elaborates the hybridization of the FFNN and COA.

### Feed forward neural network (FFNN)

The FFNNs can be highly effective for signal classification, which is the task of identifying or categorizing signals based on their characteristics. It consists of three layers namely input, hidden, and output. All the inputs for this multi-layered architecture were received through the input layers and passed the weighted input to the hidden layers to adjust its parameters for its learning purpose. It have a straightforward structure, where data flows from the input layer to the output layer without loops or cycles^51^.It was effectively work with optimal features and can able to classify the signals efficiently.

Generally, FFNN was implemented with the Levenberg-Marquardt (LM) backpropagation algorithm. It has several demerits like memory intensive, computationally expensive for large networks and datasets. Although LM excels in non-linear optimization, it struggles when the activation functions lead to overly complex or noisy optimization surfaces. Hence, we have used Cheetah Optimization Algorithm to find the optimal solution in both offline and online mode.

### Cheetah optimization algorithm (COA)

Cheetah Optimization Algorithm (COA) is one of the most important metaheuristic algorithms for problem-solving in the real-world environment. The Cheetah Optimization Algorithm (COA) is a nature-inspired optimization algorithm based on the hunting and chasing behaviours of cheetahs^51,53^.This algorithm mimics the fast, adaptive, and efficient hunting strategies of cheetahs, making it a promising approach for solving optimization problems, including those found in signal processing^51–54^. It has following key benefits with the signal processing domine like fast convergence, high precision in optimization, robustness in noisy data, efficient feature selection, and efficient signal reconstruction.

The mathematical model designed for this optimization algorithm was similar to the hunting nature of the cheetah like how it searches the prey and how it waits and catches the prey. It has the following four steps are follows:

i. searching for a prey.
ii. Waiting for a prey.
iii. Attacking the prey – This step was divided into two more steps to catch the prey efficiently. They are
  Rushing to capture the prey.
  Capturing and killing the prey.

iv. Leave the prey and return home.

#### Searching for the prey

To find prey in nearby places cheetah start to scan and search territories around it. At the time of searching the prey, the cheetah used to seek the prey in two waysFirst, it sits and scans the surrounding area; then it patrols the nearby territories, actively walking through them. Cheetah decides their searching chances depending on the coverage area of the prey condition so at the time of hunting cheetah plan to select the sit and scan mode or actively chase the prey^54^.

Whenever cheetahs try to search the prey in dense areas it used to apply the sit and search method and whenever the prey is near they use the active mode to chase the prey. During this phase position of the cheetah was denoted by the $\:{X}_{i,j}^{t}$ in which $\:I$ represents the current position $\:(\text{i}=1,\:2,\dots\:\dots\:\dots\:,\text{n})$, $\:n$ indicates number of cheetahs and $\:j\:(\text{j}=\text{1,2},\dots\:\dots\:\dots\:,\text{D})$ represents the position and arrangements of cheetah in dimension $\:D$.At the time of searching the prey, position of the cheetah $\:i\:$is updating according to the current position of the cheetah^54^. It was expressed in the below-mentioned Eq. (6).

6 $$\:{\:\:\:\:\:\:\:\:\:\:\:\:\:\:\:\:\:\:\:\:\:\:\:\:\:\:\:\:\:\:\:\:\:\:\:\:\:\:\:\:\:\:\:\:\:\:\:\:\:\:\:\:\:\:\:\:\:\:\:\:\:\:\:\:\:\:\:\:\:\:\:\:\:\:\:\:X}_{i,j}^{t+1}={X}_{i,j}^{t}+\widehat{{r}_{i,\:j}^{-1}}.{a}_{i,j}^{t}$$

Where $\:{X}_{i,j}^{t}$represents the current position and $\:{X}_{i,j}^{t+1}$ indicates the next position of the cheetah, $\:t$ indicates the hunting time, $\:{a}_{i,j}^{t}$ the step length of the cheetah and $\:\widehat{{r}_{i,\:j}^{-1}}$ represents the randomization parameter of the cheetah.

#### Waiting for the prey

During the search, cheetahs sit and wait for the prey by scanning the vision in the field. Cheetahs try to get close to their prey by lying down and hiding them to catch the prey otherwise they will wait for some time for the prey come to near^54^. This action can be expressed in Eq. (7).

7 $$\:{\:\:\:\:\:\:\:\:\:\:\:\:\:\:\:\:\:\:\:\:\:\:\:\:\:\:\:\:\:\:\:\:\:\:\:\:\:\:\:\:\:\:\:\:\:\:\:\:\:\:\:\:\:\:\:\:\:\:\:\:\:\:\:\:\:\:\:\:\:\:\:\:\:\:\:\:\:\:\:\:\:\:\:\:\:\:\:\:\:\:X}_{i,j}^{t+1}={X}_{i,j}^{t}$$

In this strategy, all the cheetahs in the dimensions were updated to improve hunting success and also to avoid premature convergence.

#### Attacking the prey

Whenever the prey is noticed by the cheetah it rushes fastly to attack the prey at full speed. At this movement, the cheetah tried to move in the direction of the prey to adjust the positions and planned to block the way of prey. Prey tried to escape from the attack to save their life, but the cheetah attacked the prey very fastly at maximum speed. During the hunting, all the cheetahs in the dimensions were adjusted it’s the position of the nearby cheetah^54^. This approach was expressed mathematically in Eq. (8).

8 $$\:{\:X}_{i,j}^{t+1}={X}_{B,j}^{t}+\:{\stackrel{\smile\!\!\!\!\!^{\smile}}{{r}_{i,j}.}}{\beta\:}_{i,i}^{t}$$

From the equations $\:{X}_{B,j}^{t}$ represents the prey’s current position, ${\stackrel{\smile\!\!\!\!\!^{\smile}}{{r}_{i,j}}}$ represents the turning factor, and $\:{\beta\:}_{i,i}^{t}$ indicates the cheetah’s interaction factor.

#### Leave the prey and return home

At the time of hunting if prey escapes from the attack then the cheetah returns to its position and starts to search the prey again. In this study, we planned to implement the hunting behaviours of cheetahs with Feed Forward Neural Network (FFNN) to determine the optimal solution for multiple solution-based problems to find out the various tasks generated by the human subjects during the study in offline and online mode.

## Results and discussions

### Experimental setup with participant performance

We conducted our experiment with 18 subjects on W-PSD features with the COA trained FFNN to determine the performance of the impaired individuals.The experiment is conducted with the following system configurations: Intel(R)Core(TM), 64 bit, Windows 10, I5 Processor, and 8 GB RAM and it was implemented in Matlab. The method was assessed by the accuracy validation metrics. It is used to calculate the amount of positively predicted samples among the total amount of all samplesand it is defined by the Eq. (9).

9 $$\:\text{A}\text{c}\text{c}\text{u}\text{r}\text{a}\text{c}\text{y}\:=\:\frac{\text{T}\text{P}+\text{T}\text{N}}{\text{T}\text{P}+\text{F}\text{P}+\text{T}\text{N}+\text{F}\text{N}}$$

Where, True positive (TP) is a count of positive class which is predicted by the model as positive. True negative (TN) is a count of negative class predicted by the model as negative. False positive (FP) is a count of negative classes predicted by the model as positive. False negative (FN) is appositive classes forecasted by the model as negative.

The classification accuracy of the 18 subjects was shown in Tables 1 and 2. From Tables 1 and 2 the highest classification of 95.90% and 95.12% were obtained by the subjects S5 and S17 from the male side and female side involved in the study. The highest maximum accuracy of 96.74% and 95.80% and minimum accuracy of 92.86% and 90.66% were attained by the male and female subjects. The next highest maximum accuracy of 95.14% and 94.43% were attained by the subjects S9 and S14 for male and female subjects. The average training time and testing time of 33.64 s, 0.76 s and 35.55 s, 0.90 s were obtained for male and female subjects with a standard deviation of 1.90 to 2.66 with an average of 2.32 and 2.18 to 2.70 with an average of 2.49. The study confirmed that standard deviation obtained from female subjects was marginally higher variability than that of the male tasks performances.

**Fig. 5:**
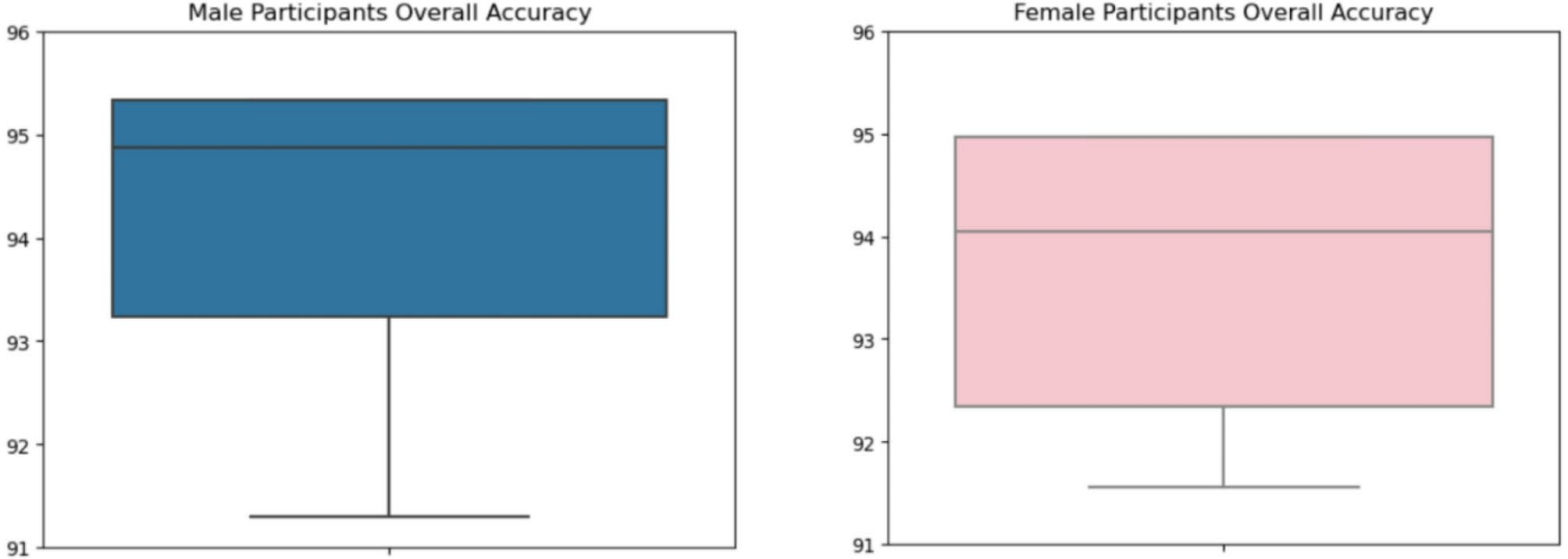
Classification Accuracy comparison between male and female participants.

Total average classification of 94.85% and 94.04% were attained for male and female subjects which was compared in Fig. 5. Subject S2 from male subjects and S10 from female subjects attained the minimum classification of 94.28% and 93.56%. From Tables 1 and 2 we analyzed that the performances of male subjects for each task were higher in contrast to female subjects. The maximum, minimum, and average classification accuracy produced by the male subjects was higher than that of the female.

**Table 1:** Classification accuracy of male participants using W-PSD features with FFNNCOA.

Subject number	Training time (sec)	Average testing time (sec)	Average accuracy (percentage)		
			Minimum	Maximum	Mean ± Standard deviation
S01	33.38	0.71	91.21	95.80	94.77 ± 2.12
S02	33.91	0.78	90.65	94.88	94.28 ± 2.56
S03	33.93	0.76	90.76	95.16	94.36 ± 2.64
S04	33.74	0.80	91.24	95.52	94.84 ± 2.35
S05	32.85	0.72	92.86	96.74	95.90 ± 1.90
S06	33.68	0.77	91.20	94.92	94.64 ± 2.11
S07	33.70	0.75	91.46	94.90	95.02 ± 2.26
S08	33.81	0.73	90.88	94.98	94.78 ± 2.36
S09	33.78	0.78	91.48	95.14	95.14 ± 2.66
Average	33.64	0.75	91.30	95.33	94.85 ± 2.32

**Table 2:** Classification accuracy of female participants using W-PSD features with FFNNCOA.

Subject number	Training time (sec)	Average testing time (sec)	Average accuracy from trails (percentage)		
			Minimum	Maximum	Mean ± Standard deviation
S10	35.34	0.93	91.08	94.92	93.56 ± 2.54
S11	35.70	0.90	91.54	94.36	93.96 ± 2.39
S12	35.68	0.92	90.73	94.69	93.82 ± 2.70
S13	35.42	0.94	91.89	95.12	93.66 ± 2.46
S14	35.56	0.91	91.71	94.78	94.43 ± 2.48
S15	35.84	0.81	90.66	95.45	94.39 ± 2.56
S16	35.43	0.90	91.90	94.96	93.70 ± 2.52
S17	35.24	0.88	92.78	95.80	95.12 ± 2.18
S18	35.78	0.92	91.82	94.72	93.78 ± 2.63
Average	35.55	0.90	91.56	94.97	94.04 ± 2.49

### Recognizing accuracy using offline test

**Fig. 6:**
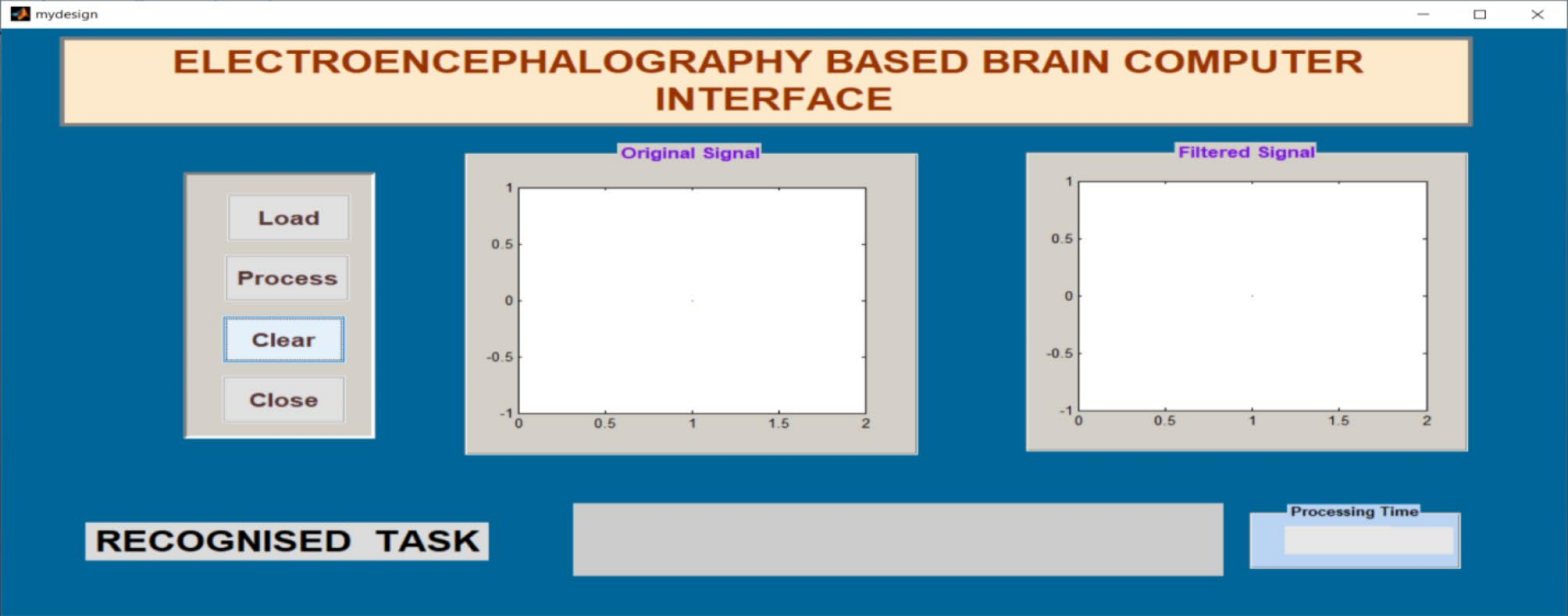
The design of GAI to verify recognizing accuracy through offline.

**Fig. 7:**
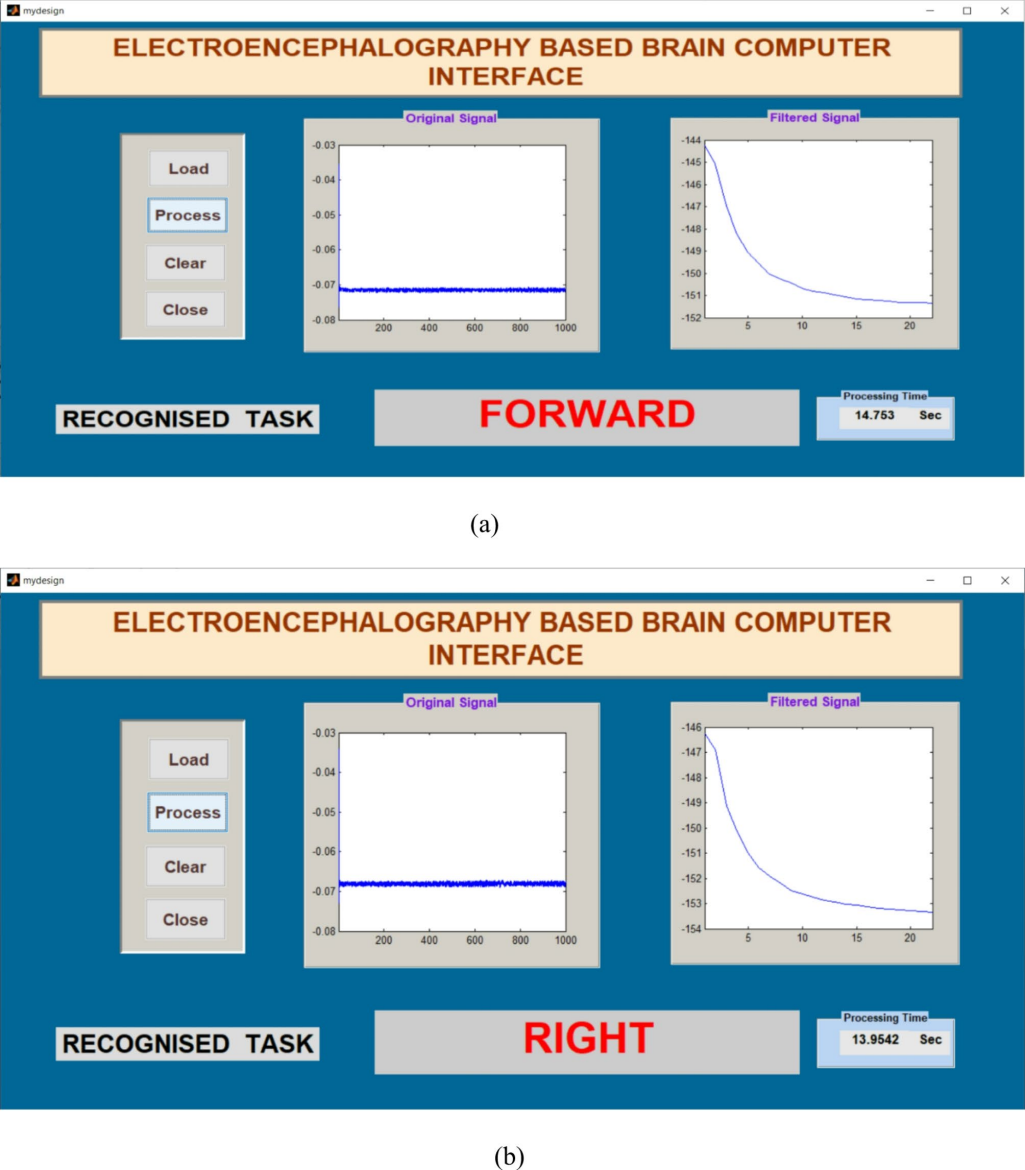

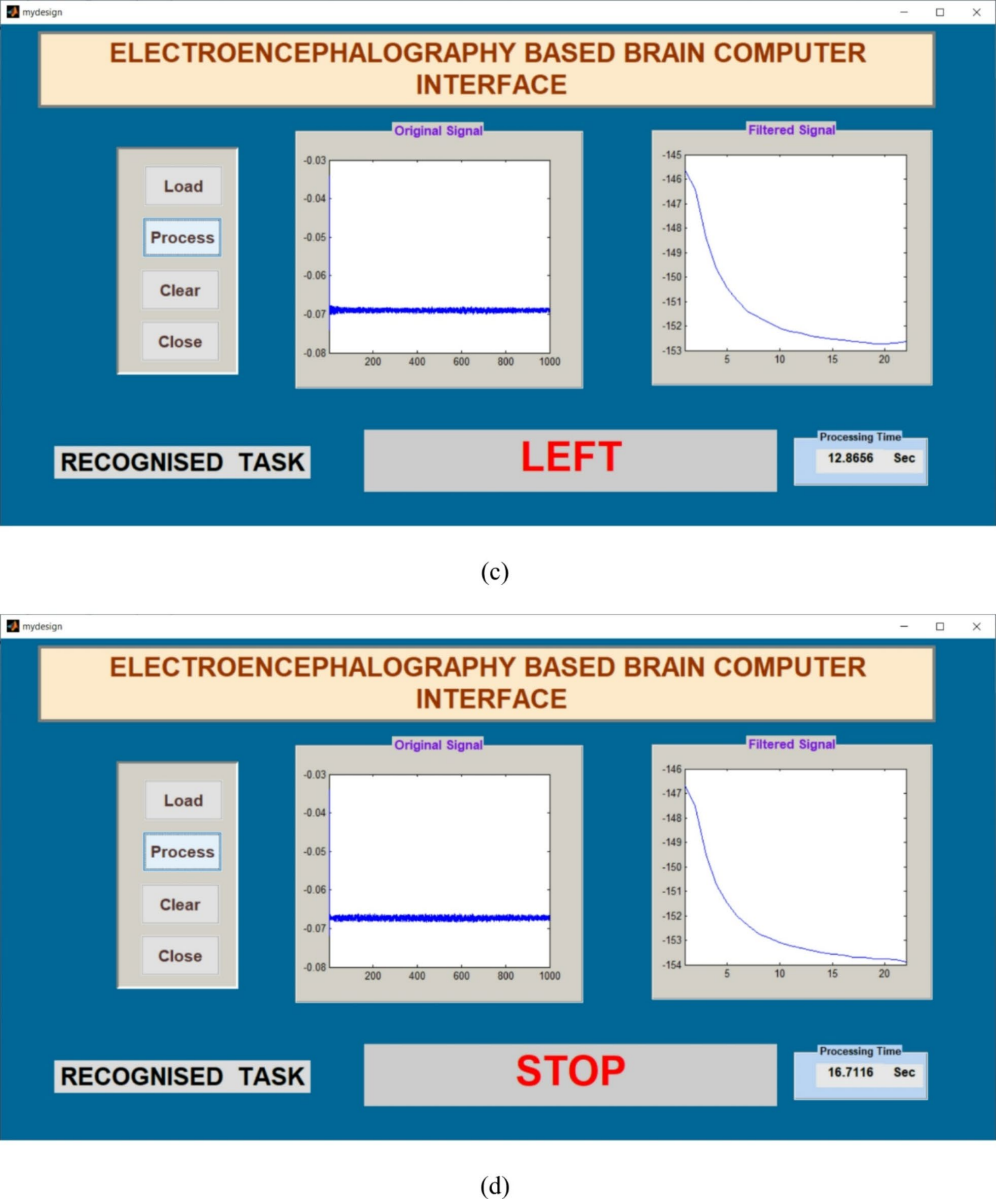
The GAI to verify tasks offline using FFNNCOA for age groups 20 to 27 for four tasks. (a) Forward, (b) Right, (c) Left, (d) Stop.

Signals obtained from participants were applied to the Graphical Application Interface (GAI) for task classification using FFNNCOA. The design of GAI and its task classification using FFNNCOA is shown in Figs. 6 and 7. The load option in GAI helps to load the participants signal from the local disk. Original signal is displayed in the first graph. The process button can able to recognize the task using FFNNCOA. After clicking process button, filter signal is displayed in the second graph. The clear button helps to clear the previous trail and close button can able to close the GAI. The processing time is displayed in the lower right corner.

**Fig. 8:**
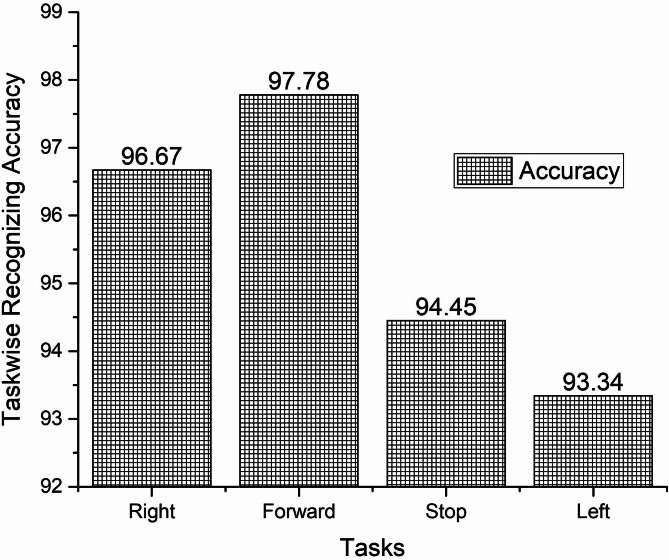
Task-wise Single Trial Recognizing Accuracy using W-PSD features for male subjects using FFNNCOA for age groups 20 to 27.

**Fig. 9:**
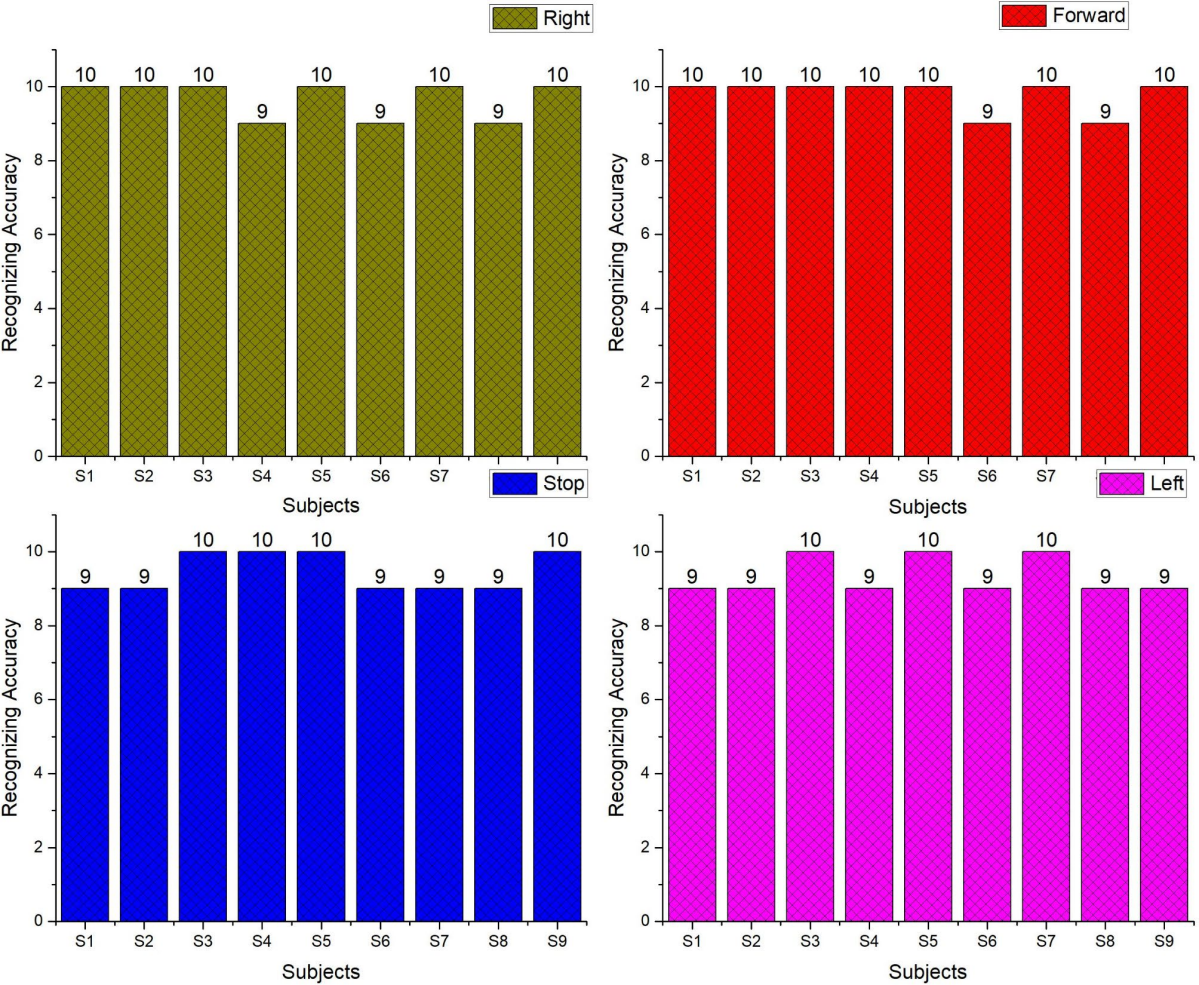
Subject-wise Single Trial Recognizing Accuracy using W-PSD features for male subjects using FFNNCOA for age groups 20 to 27.

**Fig. 10:**
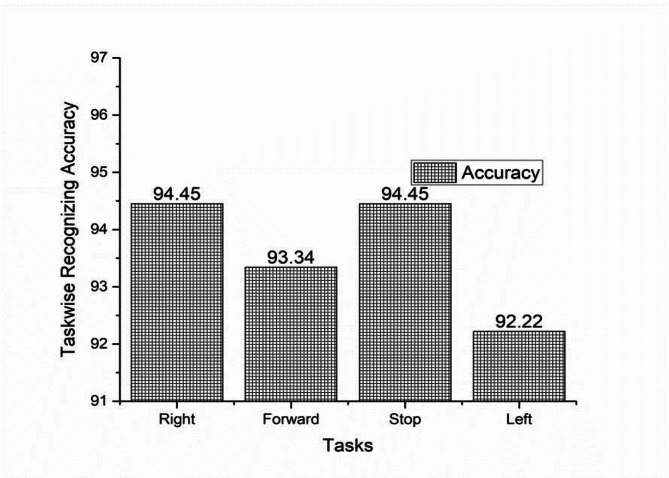
Task-wise Single Trial Recognizing Accuracy using W-PSD features for female subjects using FFNNCOA for age groups 20 to 27.

**Fig. 11:**
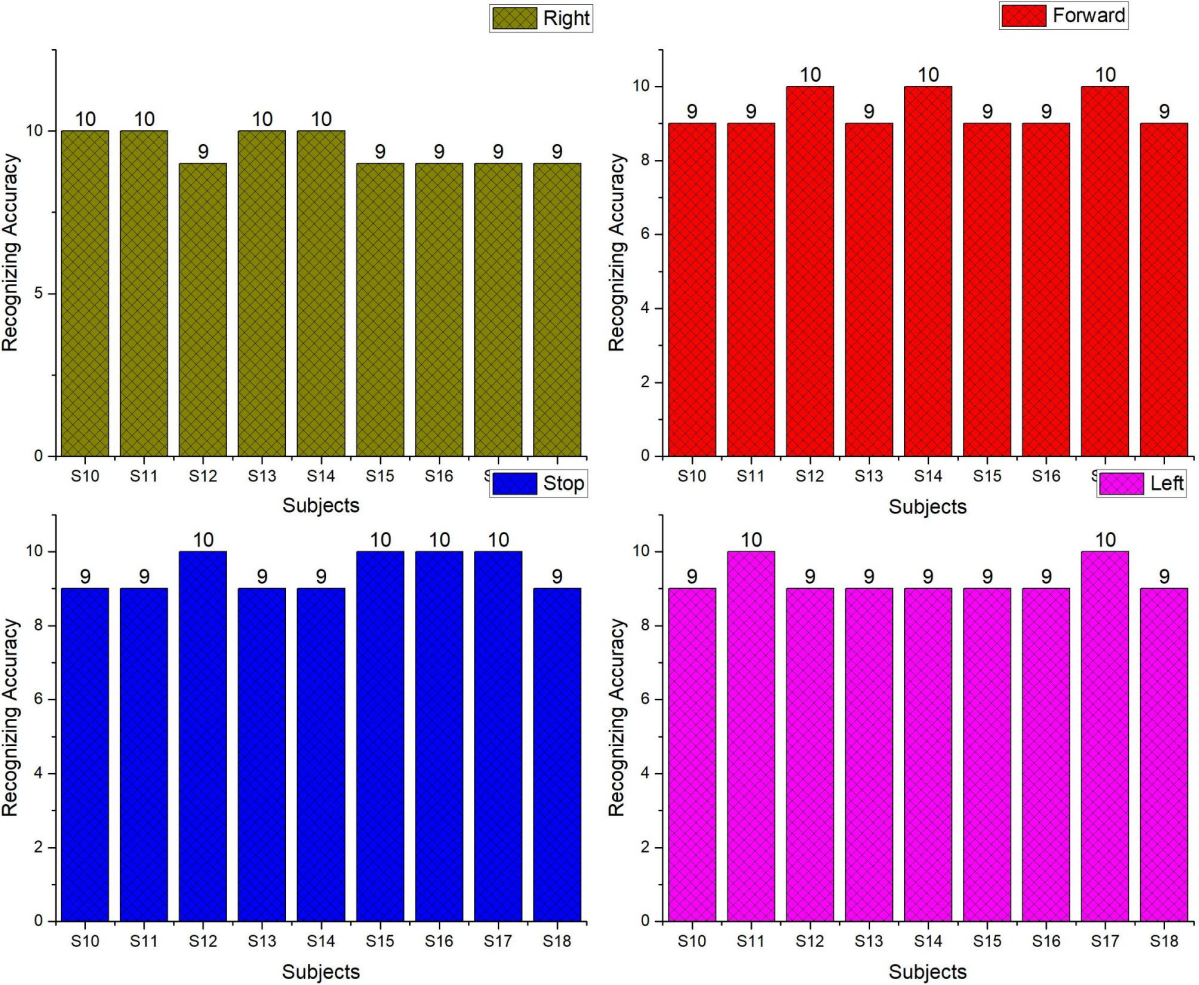
Subject-wise Single Trial Recognizing Accuracy using W-PSD features for female subjects using FFNNCOA for age groups 20 to 27.

The offline recognizing accuracy of the single trial analysis of the 18 subjects is shown in Tables 3 and 4. From Tables 3 and 4 we analyzed the individual task accuracy of 96.67% and 94.45%, 97.78% and 93.34%, 94.45%, 93.34% and 92.22% for right, forward, stop, and left tasks were shown in Figs. 8 and 10. From the individual subject wise analysis subjects S5 and S3 obtained 100% accuracy and second maximum offline accuracy of 97.50% were attained by the subjects S7 and S9. The lowest online accuracy of 90% was achieved by subject S6 from the male side which was shown in Fig. 9. From the female side, individual maximum accuracy of 97.50% was attained by subject S17. The second maximum offline accuracy of 95.00% was attained by the subjects S11, S12, S14 and S16. The lowest online accuracy of 90% was achieved by subject S18 which was shown in the Fig. 11.

Out of 360 trials, 16 and 27 trials from the male and female subjects were wrongly classified. The average offline individual subject-wise accuracy of 95.86% and 93.88% were obtained by male and female subjects. From the offline single trial analysis, the performances of male subjects were maximum compared with female subjects in terms of task and individual subject-wise. Additionally, we evaluated our study in terms of precision, recall, and F1 scores to determine the system beyond accuracy. During offline mode, it shows accuracy of 94.12%, 94.85%, and 94.48% for male participants and 93.34%, 94.04%, and 93.69% for female participants for metrics like precision, recall, and F1 scores. The result proved that more training was required for female subjects compared with male subjects.

**Table 3:** Recognizing accuracy using W-PSD features for male subjects using FFNNCOA in offline single trial analysis.

Subject	Recognizing accuracy					
	Right	Forward	Stop	Left	Wrongly classified trials	Subject wise recognizing accuracy
S1	10	10	9	9	2	95.00
S2	10	10	9	9	2	95.00
S3	10	10	10	10	0	100
S4	9	10	10	9	2	95.00
S5	10	10	10	10	0	100
S6	9	9	9	9	4	90.00
S7	10	10	9	10	1	97.50
S8	9	9	9	9	4	90.00
S9	10	10	10	9	1	97.50
Total	87	88	85	84	16	
Task wise average accuracy	96.67	97.78	94.45	93.34		

**Table 4:** Recognizing accuracy using W-PSD features for female subjects using FFNNCOA in offline single trial analysis.

Subject	Recognizing accuracy					
	Right	Forward	Stop	Left	Wrongly classified Trials	Subject wise recognizing accuracy
S10	10	9	9	9	3	92.50
S11	10	9	9	10	2	95.00
S12	9	10	10	9	2	95.00
S13	10	9	9	9	3	92.50
S14	10	10	9	9	2	95.00
S15	9	9	10	9	3	92.50
S16	9	9	10	9	2	95.00
S17	9	10	10	10	1	97.50
S18	9	9	9	9	4	90.00
Total	85	84	85	83	27	
Task wise average accuracy	94.45	93.34	94.45	92.22		

### Recognizing accuracy using online study

**Fig. 12:**
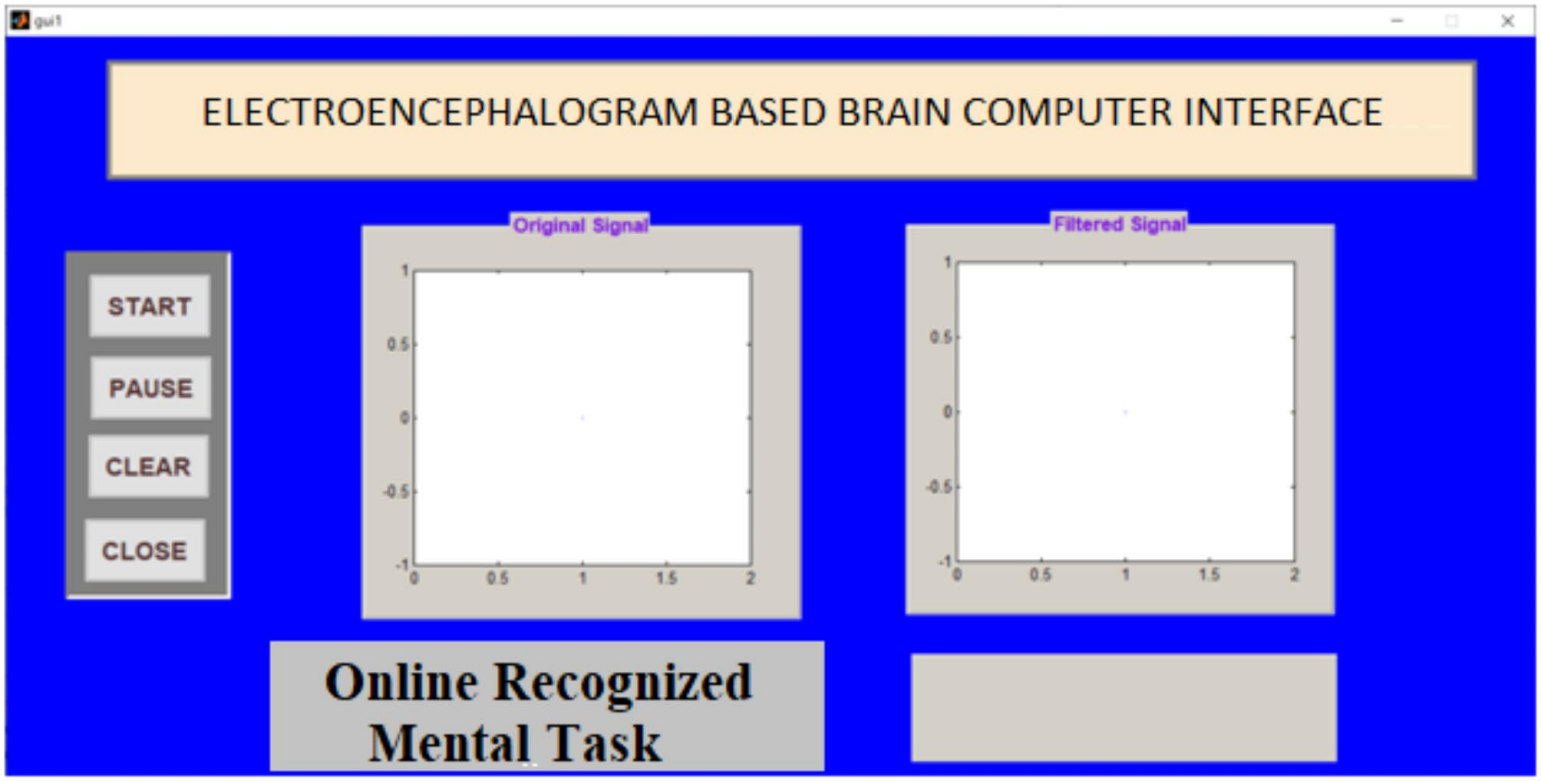
GAI to verify Recognizing Accuracy through online.

**Fig. 13:**
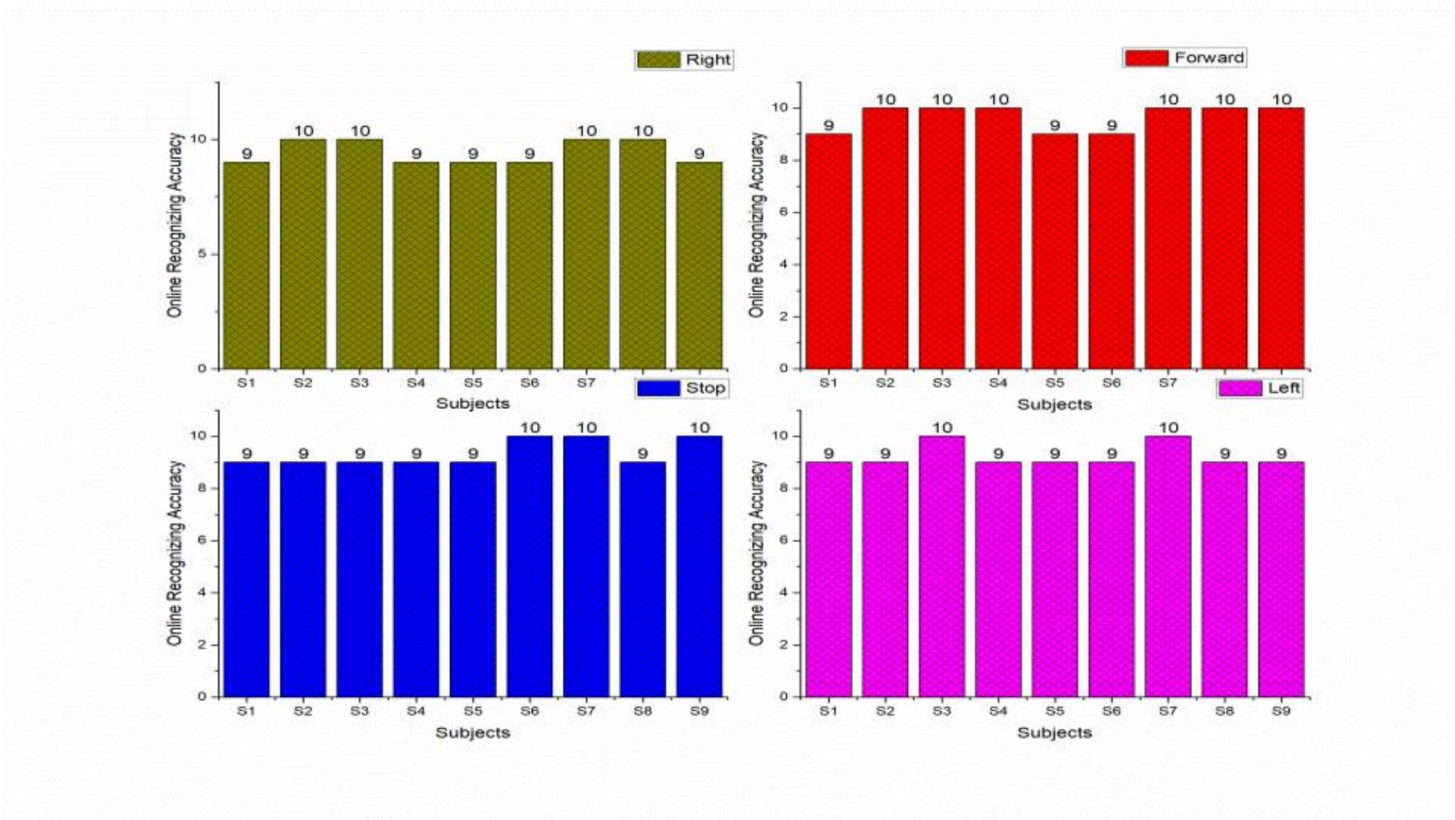
Online individual trial analysis for male subjects.

**Fig. 14:**
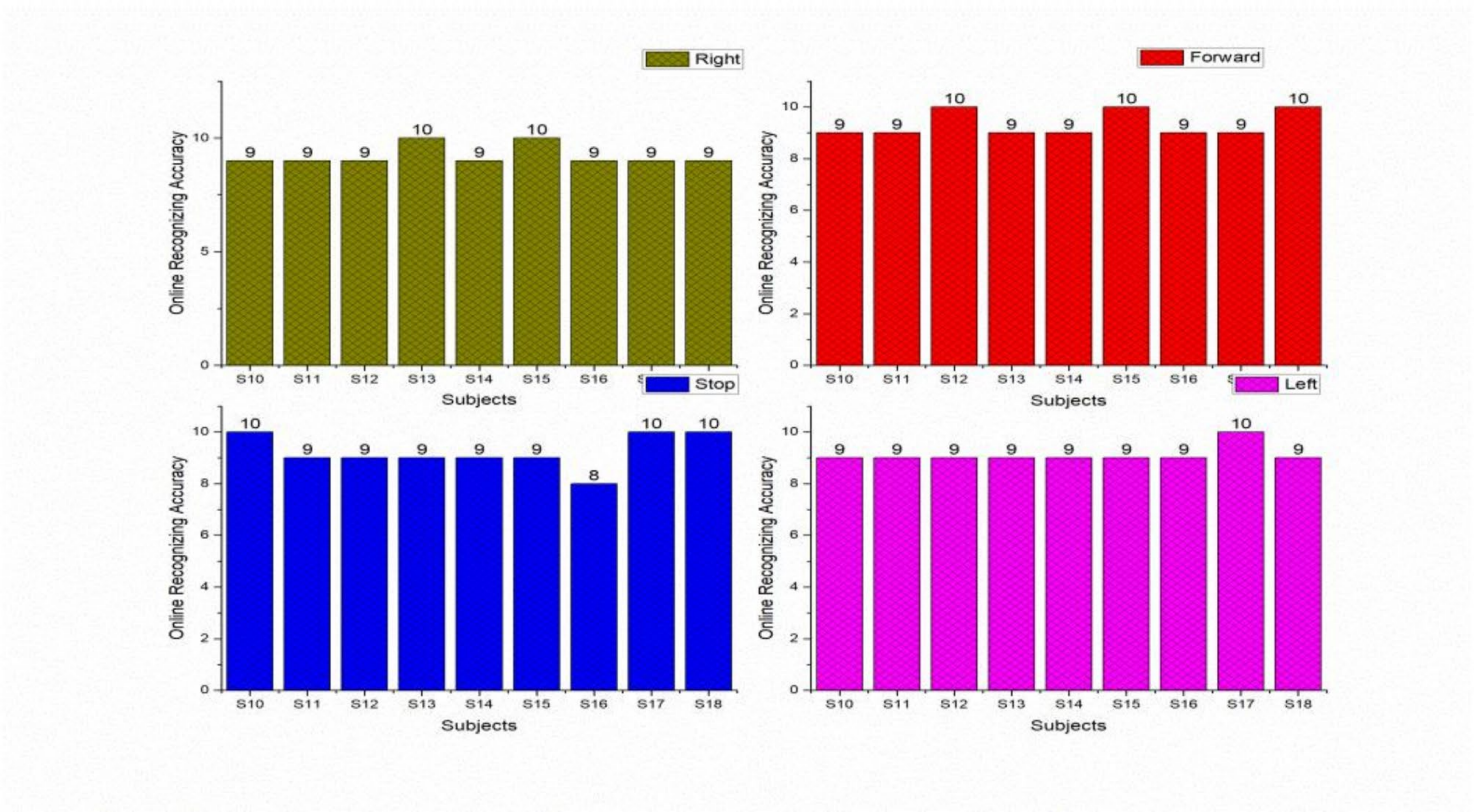
Online individual trial analysis for female subjects.

The same trained subjects were requested to perform the online tasks as per the protocols. The online signals received from the subjects were extracted and classified using the classifier and the recognized pattern was visualized with the help of Graphical Application Interface (GAI) which was illustrated in Fig. 12. GAI determined the task performed by the subjects to drive the external devices. The online recognizing accuracy of the male and female subjects was shown in the Tables 5 and 6. We obtained the maximum individual accuracy of 100% and 95.00% for S7 and S15, S17, and S18 and second maximum accuracy of 97.50% for the subjects S3 and S11, S14 minimum individual accuracy of 90.00% for male and female subjects which was shown in the Figs. 13 and 14.

**Fig. 15:**
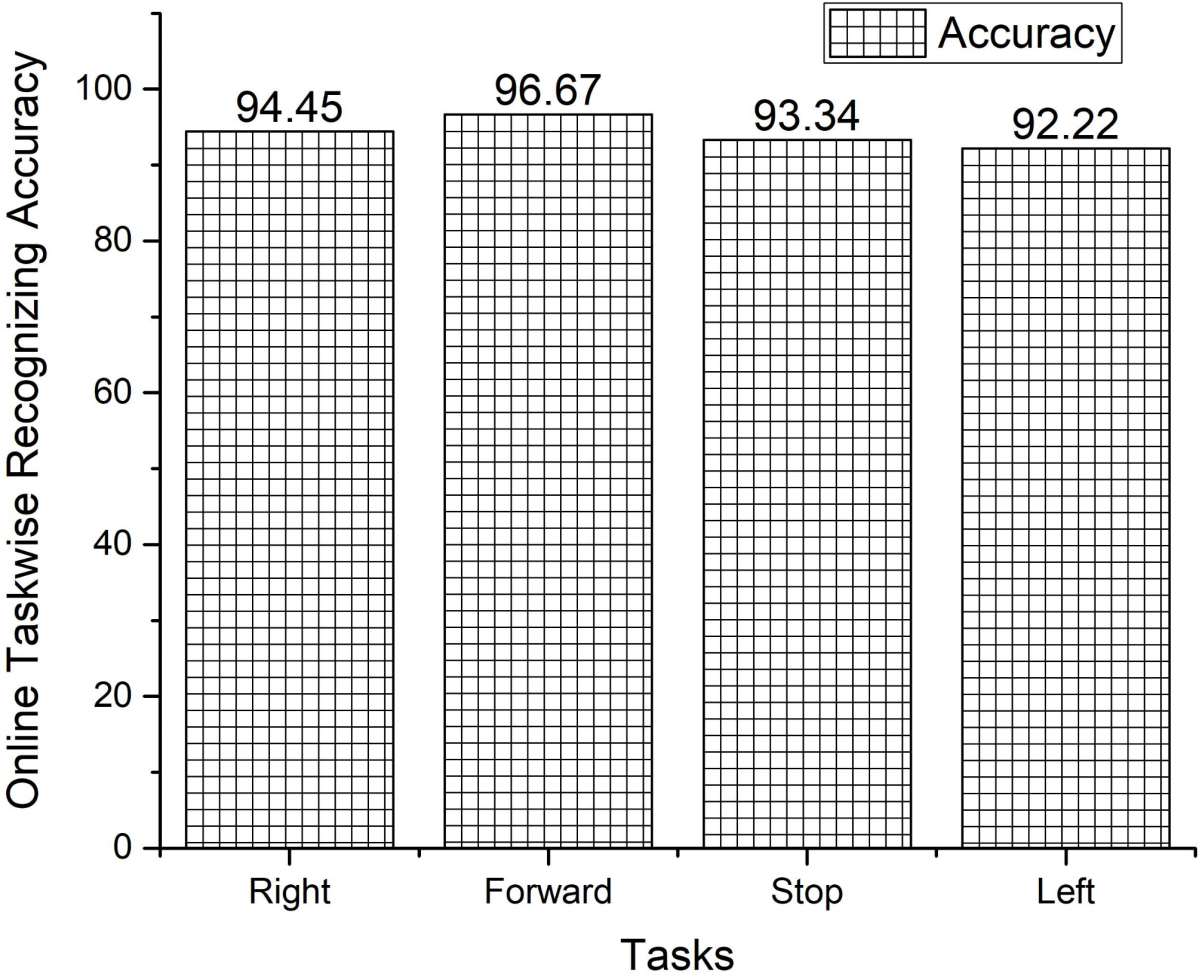
Online task-wise analysis for male subjects.

**Fig. 16:**
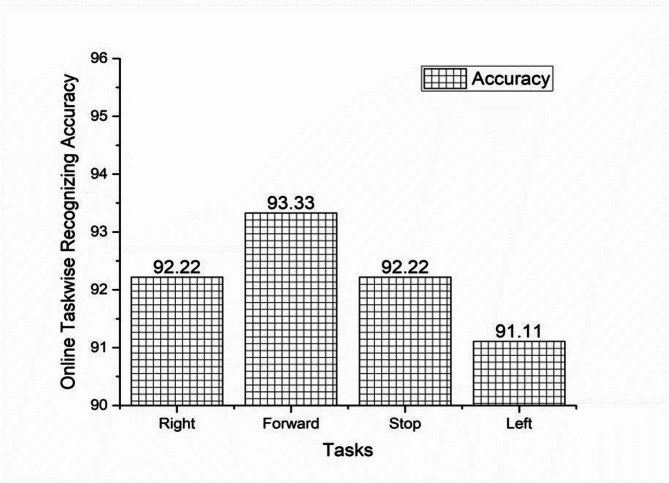
Online task wise analysis for female subjects.

Task wise analysis were conducted for all the subjects and the result were shown in the same Tables. From Tables 5 and 6 the result showed the individual online task recognizing accuracy of 94.45% and 92.22%, 96.67% and 92.22%, 93.33%, 92.22% and 92.22% and 92.22% and 91.11%, for right, forward, stop and left tasks were shown in the Figs. 15 and 16. The overall online classification accuracy varied from 90.00 to 100% and 90.00% to 95s.00% for male and female subjects. From the result, we identified that 94.17% and 92.22% of the individual trials were classified correctly in the online study and 5.83% and 7.78% of trials were wrongly classified during the online experiment for male and female subjects. During online mode, it shows accuracy of 93.78%, 94.17%, and 93.97% for male participants and 92.12%, 92.22%, and 92.17% for female participants for metrics like precision, recall, and F1 scores. These comparisons provided that performance metrics were marginally higher for offline mode compared with online mode. The study proved that all the subjects were voluntarily involved to give the best performances but some of the female subjects involved in the study were unable to switch over from one task to another task suddenly and also felt that they needed some required time to switch over from one task to another task respectively.

This hybrid approach using three electrode system simplified conventional experimental process with improved the classification accuracy without compromising accuracy and maintain computational efficiency gap between real world applications and theoretical models.

**Table 5:** Online accuracy using W-PSD features with FFNNCOA for male subject.

Subject	Online recognizing accuracy using W-PSD features with FFNNCOA				
	Right	Forward	Stop	Left	Wrongly Classified Trials
S1	9	9	9	9	4
S2	10	10	9	9	2
S3	10	10	9	10	1
S4	9	10	9	9	3
S5	9	9	9	9	4
S6	9	9	10	9	3
S7	10	10	10	10	0
S8	10	10	9	9	2
S9	9	10	10	9	2
Total	85	87	84	83	21
Online task accuracy	94.45	96.67	93.34	92.22	5.83

**Table 6:** Online accuracy using W-PSD features with FFNNCOA for a female subject.

Subject	Online recognizing accuracy using W-PSD features with FFNNCOA				
	Right	Forward	Stop	Left	Wrongly classified trials
S10	9	9	10	9	3
S11	9	9	9	9	4
S12	9	10	9	9	3
S13	10	9	9	9	3
S14	9	9	9	9	4
S15	10	10	9	9	2
S16	9	9	8	9	5
S17	9	9	10	10	2
S18	9	10	10	9	2
Total	83	84	83	82	28
	92.22	93.33	92.22	91.11	7.78

## Conclusion

The experiment was conducted with 18 subjects to determine the performances in online mode and offline mode using W-PSD features with hybrid optimization classifiers. From the study, we attained the average classification accuracy of 94.86% and 94.05%. Through offline and online analysis we obtained the average individual subject-wise recognizing accuracy of 95.56%, 93.88%, and 94.17%, 92.22% for both male and female subjects. From the study, we concluded that male subjects outperformed the female subjects in terms of accuracy. Female subjects found some difficulties in switching over from one task to another task at the time of performing the online accuracy.

### Future study

In our upcoming study, we intended to address identified limitations of our study and also planned to conduct our study in online mode with naive users in an indoor environment to check the recognizing accuracy of the developed BCI in real time.

## Data Availability

All data used in this study were available with us. The data that support the findings of this study are not openly available due to reasons of sensitivity and are available from the corresponding author upon reasonable request.
